# Accuracy and Reproducibility of Semidigital Versus Fully Digital Cephalometric Tracings Using a New Computer Program Versus Conventional Methods (Gold Standards): A Preliminary Study

**DOI:** 10.1155/bmri/8403357

**Published:** 2025-08-18

**Authors:** Farhad Sobouti, Sepideh Dadgar, Sina Namadian, Hamid Reza Bahrami Rad, Vahid Rakhshan

**Affiliations:** ^1^Orthodontic Department, Faculty of Dentistry, Mazandaran University of Medical Sciences, Sari, Iran; ^2^Orthodontic Department, Faculty of Dentistry, University of Toronto, Toronto, Canada; ^3^Mazandaran University of Medical Sciences, Sari, Iran; ^4^Department of Dental Anatomy, Dental Faculty, Azad University of Medical Sciences, Tehran, Iran

**Keywords:** cephalometrics, computerized cephalometric tracing, computerized dentistry, diagnostic accuracy, digital dentistry, digital imaging, landmarking, manual tracing, orthodontics, reliability, reproducibility

## Abstract

**Introduction:** Cephalometric tracing can be done either conventionally or using computers. Digital dentistry and digital orthodontics have considerably facilitated procedures. Still, their diagnostic accuracy needs assessment. Many orthodontic programs have been developed for this purpose. The efficacy and reliability of such software are usually compared with the conventional method (gold standard). We used novel and more stringent methods of assessment to test a program in this regard.

**Methods:** This study was performed on 10,302 tracing evaluations within 101 cases. Lateral cephalograms of 101 patients were landmarked using two methods (on paper vs. on a computer screen) and traced using three methods (completely conventionally [gold standard]; landmarks were identified on paper, but measurements were calculated by computer; landmarks were identified on the computer screen, and measurements were calculated by the computer program). A total of 15 landmarks and 17 cephalometric tracing measurements were determined via the abovementioned methods. The tracing errors were defined as differences between each pair of tracing methods, as well their absolute values (a total of 6 different tracing errors). Intraclass correlations were calculated for tracing values. Tracing errors were compared with the value 2, as the clinically acceptable range. However, they were also compared with the values zero as well as one hundredth of the mean of gold standard (as a more conservative value), using a one-sample *t*-test (*α* = 0.05).

**Results:** All tracing errors were smaller than the clinically acceptable limits. Moreover, most simple errors were close to zero, and/or below the criterion of 1/100 of the mean of the gold standard. Furthermore, the more difficult error tests, that is, the directionless absolute errors, were all below 2; additionally, they were either below the 1/100 of absolute of the gold standard means or at the level of those means. Finally, the intraobserver reliabilities were high. All the 102 simple errors and absolute errors (on 101 lateral cephalograms) were significantly below 2 (*p* < 0.0005, clinically acceptable).

**Conclusions:** The accuracy was appropriate. Of the 51 simple tracing errors, only 9 were significantly greater than zero, and many of them were below or at the level of 1/100 of the gold standard means. All the directionless (absolute) errors were significantly greater than zero. However, in the case of those calculated as “absolute value of (gold standard subtracted by fully digital method),” all errors were below or at the level of 1/100 of the absolute of gold standards' means. The intraobserver reliabilities were high.

## 1. Introduction

For long, conventional two-dimensional radiographic techniques have been utilized effectively for orthodontic diagnosis and treatment [1]. Cephalometric tracing is key to orthodontic and orthognathic treatments, being heavily used in diagnosis, treatment planning, and treatment outcome/prognosis evaluation [2]. Nowadays, cephalometric tracing is performed using both conventional and computerized methods [3, 4]. Conventional tracing, the gold standard, is performed using acetate paper and pencil on conventional cephalographs [5, 6]. Computerized tracing uses cephalometric software programs to open digital cephalographs; later, landmarks are identified on digital cephalograms by the clinician, and these orthodontic landmarks are used to complete geometrical cephalometric tracing procedures [7]. In the conventional method, performing so many slow measurements on paper one by one is painstakingly time-consuming [8].

This can be eliminated by cephalometric programs that enable the orthodontist to do cephalometric tracings in almost no time [6, 9, 10]. Such computer programs can facilitate cephalometric tracing through the advent of many features such as digital zoom, brightness enhancement, contrast enhancement, very accurate linear and angular measurements, fast storage and loading, as well as eliminating the need to draw lines (because cephalometric lines are automatically drawn between landmarks) [11, 12]. Nevertheless, there can be errors. Cephalometric errors can be categorized into projection errors (acquisition of a cephalograph), landmark identification errors, and measurement errors [13, 14].

Nevertheless, such software programs might be prone to errors [9, 15]. For example, since the resolution of digital cephalographs is usually lower than the resolution of the conventional cephalographs, it is possible that some errors happen during the landmarking stage, which might translate into errors in tracing [11, 12, 16, 17]. This calls for properly testing new cephalometric software available in the market to ensure their accurate procedure of digital tracing [10, 15].

Previous results have been controversial; still, earlier studies have indicated that cephalometric programs can be reliable at least in terms of some cephalometric measurements, if not all of them [16, 18–21]. In this regard, to the best of our knowledge, all studies that have assessed errors have ignored the fact that the direction of errors (negative or positive) may cancel out each other, leading to underestimated errors [16, 18–21]. Furthermore, each new program created in this regard needs to be tested before mass use. A novel computer program has been developed by the authors for the computerization of lateral cephalometric analysis (not commercial; merely for research purposes). Nevertheless, its accuracy has not been examined yet. Therefore, it is necessary to first assess its reproducibility and accuracy compared to the gold standard, which is the conventional method of cephalometric tracing. This study is aimed at examining this.

For this purpose, we conducted a study on 101 lateral cephalographs to be first landmarked both conventionally and via the new computer program, and then traced them once on paper (fully digital), once on the computer, and once as a semidigital method (details below).

When comparing software measurements with the respective gold standards, the program measurements can be larger or smaller than the gold standard. Both of these are considered errors, but positive and negative errors, respectively. In such a pool of errors with different signs, calculating the average of these errors will result in a value that tends to be closer to zero, because negative and positive values may partially cancel each other out. Although this form of error is valuable, its average value might be exaggeratedly small and thus underestimate the extent of errors. Therefore, author VR also defined a new, directionless tracing error. This form of error disregards whether the software measurements are larger or smaller than the respective gold standard; it only concerns itself with the absolute value of the difference. In other words, this definition of error disregards the “direction” or sign of each error by calculating its absolute value. The merit of this method is that when the average of errors is being computed, there is no direction of error (i.e., smaller or larger), but there is only the extent of difference as a positive number. Mathematically, the errors in different directions will no longer have different signs and, hence, cannot cancel each other out. In other words, the average error would be farther from zero, compared to when the errors have both negative and positive signs. This form of tracing error yields greater average error values compared to the simple errors that have both negative and positive errors. Therefore, such absolute error values are more conservative, as their averages tend to be larger, at the expense of losing information about the directions of errors. It should be noted that both methods (directionless and conventional) have merit, have their own disadvantages, and present different information. The conventional method is valuable in that it presents relevant information about errors and also their directions, but at the expense of lower sensitivity. On the other hand, the new error provides higher sensitivity and larger average errors, but it truncates information about the direction of errors.

The primary null hypothesis was a lack of any significant difference between the landmarking and/or tracing using this program compared to the gold standard or another reference (detailed below). We defined two different concepts of tracing errors: simple errors always reported before and directionless errors not reported before (to the best of our knowledge). We compared these tracing errors with the values 2 mm or 2° as clinically acceptable limits [16, 20–23].

Furthermore, another new method was presented in this study. All previous studies have considered tracing errors about 2 mm or 2° or smaller than the gold standard as clinically acceptable [16, 20–23]. This kind of error is not without drawbacks, because it is fixed and does not take into account the tracing measurement in question. While tracing measurements may vary greatly, this error threshold is fixed at 2 mm and 2° and does not take into account the extent of the measurement itself. It is reasonable to expect larger measurements to have larger errors and smaller measurements to have smaller errors. Nevertheless, the 2-mm/2° threshold does not consider this and, thus, can be too large for tracing measurements that are inherently small and too small for measurements that are inherently large. Therefore, author V.R. defined a new threshold that flexibly changes depending on the measurement itself, not to mention that it can also be much more stringent than the conventional fixed value for small measurements. We propose that the error should not be greater than 1/100 of the gold standard value. Consequently, if a measurement is inherently small (for example, 10 mm), this new “acceptable error threshold” would be small accordingly (for example, 10/100 = 0.1 mm); this example threshold of 0.1 would be much smaller and much more conservative than the 2-mm error threshold. Consequently, in our study, cephalometric tracing errors (and directionless tracing errors) were tested against one hundredth of the mean of the gold standard or in terms of directionless errors. We did not limit ourselves to this new error threshold either; we also compared the errors with a third error threshold, that is, the value zero, as the ideal extent of “no error.”

## 2. Materials and Methods

This study was performed on 10302 evaluations: 102 assessments per case, within 101 cases. This was an explorative, experimental, diagnostic pilot study on 101 archival cephalographs, including three phases of landmarking of cephalometric landmarks and cephalometric tracings using three methods, within 101 digital cephalographs. The images in use were all archival and taken retrospectively and merely for therapeutic purposes and not for any research purposes. Therefore, no x-ray was emitted to any patient due to this study. There were no humans involved in this retrospective study; therefore, written consent was not applicable. The study protocols of the theses, a part of which this pilot study was, were approved by the Research Committee of the Mazandaran University of Medical Sciences, Sari, Iran as two dental theses registered with Numbers 1400: 4115 and 1400:4123 and ethics approval codes IR.MAZUMS.REC.1399.879 and IR.MAZUMS.REC.1399.880.

The inclusion criteria were a high quality of digital lateral cephalographs and a resolution of 1331 pixels × 1847 pixels. The cephalograms were taken from the archives of two radiology centers operating the same device model. No modifications were made to the brightness, contrast, or other settings of the images.

### 2.1. Sample Size

This was a pilot study; therefore, no sample size calculation was in place. The sample size was determined as greater than the size of the most recent studies on the reproducibility of cephalometric programs. Post hoc power calculations showed very high powers (higher than 90%–99% in most cases).

### 2.2. Experiments

The experiments were performed by a calibrated last-year dental student in three diagnostic phases: (1) the gold standard in which both landmarking and tracing were performed on the paper using conventional tools (paper-printed cephalograms, pencils, rulers, and conveyors). (2) Semidigital: The landmarking stage was done conventionally on paper-printed cephalograms; the coordinates of the landmarks were obtained. Tracings were not done on the paper; instead, the coordinates of the landmarks were inserted into the experimental orthodontic software program in order to calculate the tracing measurements. (3) Fully digital: Both the landmarking and tracing steps were done on digital images shown on the experimental computer screen, within the software environment and using the program's own tools.

1. Gold standard: Both landmarking and tracing procedures were done manually on paper. Cephalographs were printed in actual size to be landmarked and traced manually on acetate paper using H2 pencil by a trained last-year dental student. The 15 identified cephalometric landmarks were:
  i. Nasion (N). The deepest point between the nose and forehead on the midline.
  ii. Orbitale (Or). The most anterior and inferior point on the infraorbital rim
  iii. Sella (S). The midpoint of the pituitary fossa (sella turcica).
  iv. Pogonion (Po). The most anterior point on the mandibular symphysis.
  v. A point: The most concave point on the anterior maxilla.
  vi. B point: The most concave point on the anterior mandible.
  vii. Pogonion (Pog). The most anterior point on the skeletal chin on the midsagittal plane.
  viii. Gnathion (Gn). Point located perpendicular on the mandibular symphysis midway between menton and pogonion.
  ix. Gonion (Go). The most inferior posterior point on the mandibular angle.
  x. Maxillary 1i: The incisal edge of the maxillary central.
  xi. Mandibular 1i: The incisal edge of the maxillary central.
  xii. Maxillary 1a: The apex of the maxillary central.
  xiii. Mandibular 1a: The apex of the mandibular central.
  xiv. Maxillary 6c: The cusp of the maxillary first molar.
  xv. Mandibular 6c: The cusp of the mandibular first molar.

Of the two cephalometric analyses of Downs and Steiner, 17 cephalometric measurements were selected to be calculated. These were: Facial angle, convexity, A-B plane, MP angle, Y axis, the cant of occlusal plane, interincisal angle, L1-OP angle, L1-MP, U1 protrusion, SNA, SNB, ANB, OP-SN, MP-SN, U1-NA, and L1-NB. No more than 10 cephalographs were landmarked and traced a day, in order to prevent human errors caused by fatigue.

2. Semidigital method: The coordinates of the landmarks identified in the gold standard phase were identified in mm. They were entered into the experimental program. The program used geometric calculations to compute the cephalometric measurements listed above.
3. Fully digital method: The digital cephalographs were fed to the experimental software in JPEG format. In the software environment, first, the size and measurements were calibrated on the cephalographs using the cephalostat ruler. Then, cephalometric landmarks were identified on cephalographs by the same operators on a computer screen with a resolution of 1360 × 768 (SyncMaster, Samsung, Korea). The program had functions for digitally zooming in and out of the images. The user identified the landmarks, and then the program calculated the coordinates of each landmark and measured the above-mentioned 17 cephalometric measurements based on the landmarks' coordinates. No more than 10 cephalographs were examined a day in order to prevent fatigue or human errors.

### 2.3. Tracing Errors

Tracing errors were calculated as two different concepts:

#### 2.3.1. Simple Tracing Errors

These errors were calculated as a particular cephalometric measurement given in a reference method (the more conventional one) minus the tested method (done using more modern technologies). It included three errors for each of the 17 tracing measurements:

1. Gold standard—semidigital
2. Gold standard—fully digital
3. Semidigital—fully digital

In the above errors, a positive error meant a larger measurement in the reference method compared to the tested method, while a negative error meant a smaller measurement in the reference method compared to the tested method. Therefore, the obtained mean errors and their standard deviations (SDs) could convey information regarding the error extent, taking into consideration the direction of the error (i.e., greater or smaller than the reference). In this type of error, it is possible that various negative and positive errors of a given measurement cancel out each other, resulting in mean errors close to zero.

#### 2.3.2. Directionless (Absolute) Tracing Errors

This error was calculated for each measurement of each patient as the absolute value of the abovementioned simple errors:

1. Absolute of (gold standard—semidigital)
2. Absolute of (gold standard—fully digital)
3. Absolute of (semidigital—fully digital)

This error type disregards the direction of the error in each patient and emphasizes the extent of the error. Since all the directionless error values are positive, the mean directionless errors are anticipated to be larger than the mean simple errors. To the best of our knowledge, this error has not been evaluated before.

An ideal (lack of) tracing error is when the averages of both simple and especially absolute errors are close to zero. Clinically acceptable errors are suggested as below 2 mm or 2° [16, 20–23]. However, we used much more stringent criteria for this assessment: we calculated 1/100 of the mean of the gold standard for each tracing measurement and considered it as the acceptable limit. For the directionless errors, we considered the absolute of the mean of the gold standard as the acceptable threshold.

### 2.4. Statistical Analysis

The intraobserver agreement for each cephalometric measurement calculated with the three methods was calculated for each of the 17 measurements as the Cronbach's alpha.

Descriptive statistics and 95% confidence intervals (CIs) were calculated for the measurements and tracing errors. Tracing errors (both directionless and simple errors) were compared with the constant value 2 as the acceptable clinical error for cephalometric tracing error [16, 20–23].

However, we also used much more stringent criteria as well: The tracing errors were also compared with the value 0 using the one-sample *t*-test. If this comparison was significant, they were also compared with 1/100 of the mean value of the gold standard (or in the case of the absolute errors, with the absolute value of the gold standard's mean), as a trivial nonzero error. The software in use was SPSS 25 (IBM, Armonk, New York, United States). The level of significance was set at 0.05.

## 3. Results

**Figure 1:**
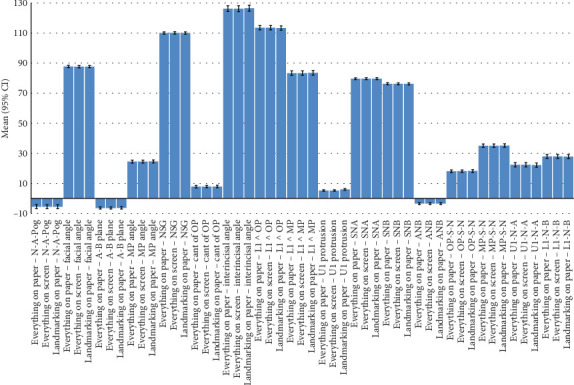
Bar charts showing means and 95% CIs for mean tracing values. The number of samples per bar is 101 (*n* = 101). Everything on Paper refers to the fully conventional gold standard, where both landmarking and tracing have been performed conventionally on the tracing paper. Landmarking on Paper refers to the semi-digital method, in which landmarking is done on paper, and then landmark coordinates are used by the program to calculate the measurements. Everything on Screen refers to the fully digital method, in which the landmarking was done by the operator on the screen, and from their coordinates, the software calculated the measurements.

There was no missing data.  Table 1 and Figure 1 compare descriptive statistics for tracing values, which were quite close to each other. The ICC showed a perfect intraobserver agreement for all the 17 cephalometric measurements (all Cronbach's alpha values ≥ 99.5%, *p* < 0.0005).

**Figure 2:**
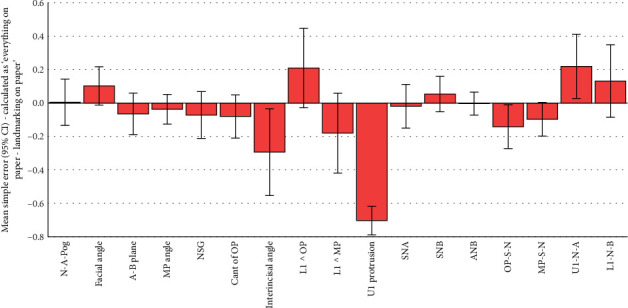
Means and 95% CIs for simple errors calculated as the gold standard (all on paper) minus semi-digital (landmarking on paper but measurements using the software). The number of samples per bar is 101 (n = 101). Everything on paper refers to the fully conventional gold standard, where both landmarking and tracing procedures have been performed conventionally on the tracing paper. Landmarking on paper refers to the semi-digital method, in which landmarking is done on paper, and then landmark coordinates are used by the program to calculate the measurements.

**Figure 3:**
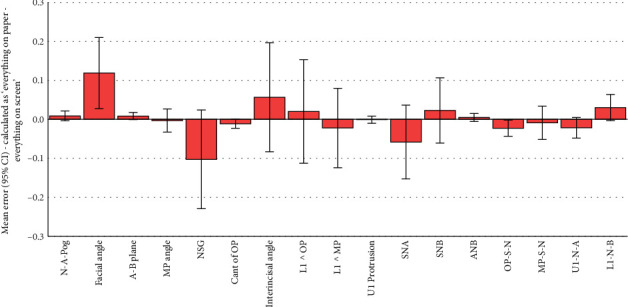
Means and 95% CIs for simple errors calculated as the gold standard (all on paper) minus fully digital (both landmarking and measurements done manually on the computer). The number of samples per bar is 101 (*n* = 101). Everything on paper refers to the fully conventional gold standard, where both landmarking and tracing have been performed conventionally on the tracing paper. Everything on screen refers to the fully digital method, in which the landmarking was done by the operator on the screen, and from their coordinates, the software calculated the measurements.

**Figure 4:**
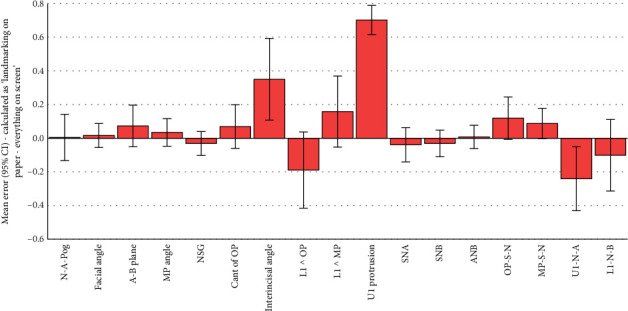
Means and 95% CIs for simple errors calculated as semidigital (landmarking on paper but calculations by computer) minus fully digital (both landmarking and measurements done manually on the computer). The number of samples per bar is 101 (*n* = 101). Landmarking on paper refers to the semidigital method, in which landmarking is done on paper, and then, landmark coordinates are used by the program to calculate the measurements. Everything on screen refers to the fully digital method, in which the landmarking is done by the operator on the screen, and from their coordinates, the software calculates the measurements.

**Figure 5:**
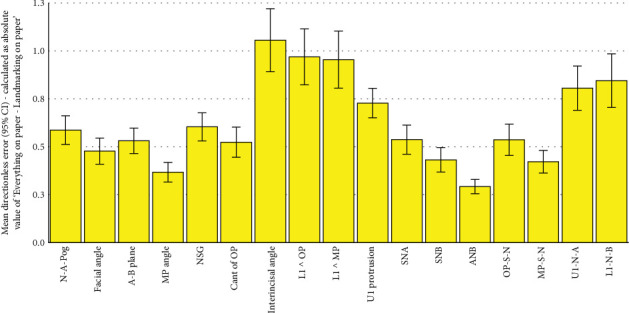
Means and 95% CIs for directionless errors calculated as the absolute value of the gold standard (all on paper) minus semidigital (landmarking on paper but measurements using the computer). The number of samples per bar is 101 (*n* = 101). Everything on paper refers to the fully conventional gold standard, where both landmarking and tracing have been performed conventionally on the tracing paper. Landmarking on paper refers to the semi-digital method, in which landmarking is done on paper, and then, landmark coordinates are used by the program to calculate the measurements.

**Figure 6:**
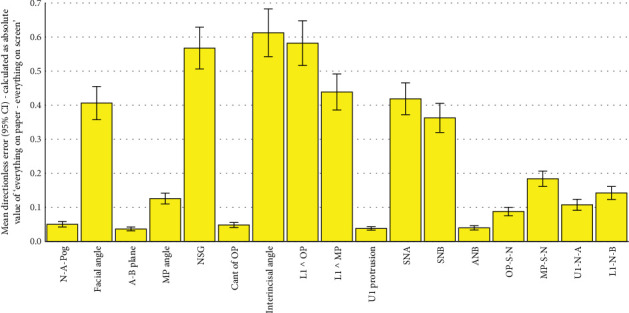
Means and 95% CIs for directionless errors calculated as the absolute value of the gold standard (all on paper) minus fully digital (both landmarking and measurements done manually on the computer). The number of samples per bar is 101 (*n* = 101). Everything on paper refers to the fully conventional gold standard, where both landmarking and tracing have been performed conventionally on the tracing paper. Everything on screen refers to the fully digital method, in which the landmarking was done by the operator on the screen, and from their coordinates, the software calculated the measurements.

**Figure 7:**
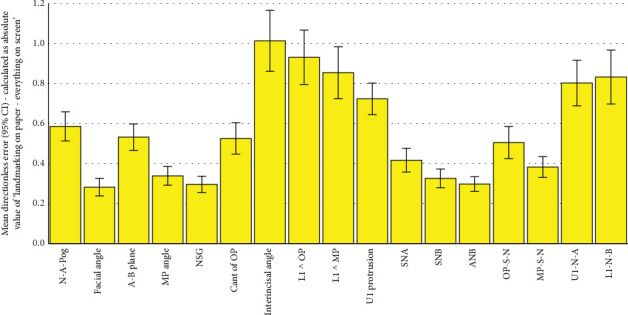
Means and 95% CIs for directionless errors calculated as the absolute value of semidigital (landmarking on paper but calculations by computer) minus fully digital (both landmarking and measurements done manually on the computer). The number of samples per bar is 101 (*n* = 101). Landmarking on Paper refers to the semidigital method, in which landmarking is done on paper, and then landmark coordinates are used by the program to calculate the measurements. Everything on Screen refers to the fully digital method, in which the landmarking is done by the operator on the screen, and from their coordinates, the software calculated the measurements.

All the 102 simple and absolute tracing errors were significantly smaller than 2 mm or 2° as the clinically acceptable error range (all 102 *p* values < 0.0005; one-sample *t*-test; Tables 2, 3, 4, 5, 6, and 7; and Figures 2, 3, 4, 5, 6, and 7).

Of the 51 simple tracing errors, only 9 were significantly greater than zero (Tables 3, 4, and 5 and Figures 3, 4, and 5). Of these 9 errors, 4 occurred when simple tracing errors were calculated by subtracting the semidigital results from the gold standard. The follow-up comparisons of the results of these four variables with the 1/100 of gold standard showed that Interincisal Angle (although significantly greater than zero) was also significantly different from 1/100 of the gold standard. The same also applied to the protrusion of the maxillary incisor and OP-S-N, but not U1-N-A, which was not different from 1/100 of the gold standard (Table 3). Of the 9 simple errors significantly different from zero, 2 happened when simple tracing errors were calculated by subtracting the fully digital results from the gold standard. One of these 2 was facial angle: although the error in this angle was significantly greater than zero, the error was much smaller than 1/100 of the gold standard. The other variable (OP-S-N) was significantly different from both zero and 1/100 of the gold standard (Table 4). Of the 9 simple errors significantly different from zero, 3 occurred when simple tracing errors were calculated by subtracting the fully digital results from semidigital results. Of these 3, 2 were also significantly larger than the 1/100 of gold standard (the protrusion of the maxillary central and U1-N-A). Nevertheless, one of them (interincisal angle) was much smaller than the 1/100 of the gold standard.

All the 51 directionless tracing errors were significantly greater than zero (Tables 5, 6, and 7 and Figures 5, 6, and 7). The follow-up comparisons with the absolute values of 1/100 of gold standards showed that many of these errors were either insignificantly different from the absolute of 1/100 of the gold standard or significantly smaller than it (very small directionless errors). These favorable directionless errors were facial angle, NSG, interincisal angle, L1 ^ OP, L1 ^ MP, SNA, and SNB in the errors calculated as the absolute of semidigital results subtracted from the gold standard (Table 5). When evaluating the directionless errors calculated as the absolute value of fully digital subtracted from the gold standard, all directionless errors became favorably smaller than or similar to the absolute of 1/100 of the gold standard (Table 6). The favorable directionless errors calculated as the absolute value of fully digital results subtracted from semidigital comprised facial angle, NSG, interincisal angle, L1 ^ OP, L1 ^ MP, SNA, SNB, and MP-S-N (Table 7).

## 4. Discussion

Several previous articles have compared conventional cephalometric analysis versus different cephalometric analysis software programs in terms of procedure time, accuracy, reliability, and repeatability of software results [16, 18]. Our study was, however, on a new program not assessed before, and it examined several variables on numerous cephalographs. This study used two new assessment methods, that is, the more stringent directionless errors and the relative 1/100 error. Besides, we tried to improve our evaluations by examining both landmarking and tracing errors, and extending the tracing methods to two fields of semidigital and fully digital fields. The findings of this study showed promising results in terms of simple tracing errors and even much more stringent absolute (directionless) tracing errors. All of these were smaller than 2 mm or 2° (which is the acceptable error range) [16, 20–23]. Moreover, the fully digital tracing method caused all tracings to become significantly below or at the 1/100 of the absolute value of the gold standard. Besides the high accuracy, all reliability indices became perfect, that is, 100% intrarater agreements. On the other hand, the semidigital method (in which landmarking was done on paper but tracing was done using landmark coordinates) found to be not as accurate as either the gold standard or the fully digital cephalometric tracing (although still clinically acceptable). Our perfect ICC values were similar to previous studies that had calculated high ICCs [16].

In this study, we tried to evaluate the experimental software using methods that were more stringent than previously used methods. It is suggested that clinically speaking, errors within 2° for angular measurements or 2 mm for linear ones might probably not make a difference in therapeutic outcomes and thus are clinically negligible [16, 20–23]. In this regard, our results were appropriate. However, we did not limit ourselves to these suggested thresholds. Instead, we also selected more stringent criteria for assessing the digital software program, i.e., the no-error zero threshold and the 1/100 of the gold standard values or absolute gold standard values, which were much smaller than 2° or 2 mm. Despite the more stringent criterion used in this study, we still found that tracings done using this computer program yielded reasonably small simple errors as well as absolute (directionless) errors that all were at or below the stringent threshold being 1/100 of the gold standards' absolute means. Not to mention that these errors were already smaller than the clinically accepted levels of 2 ° or 2 mm. Future studies are warranted to use this method together with the more relaxed conventional method of error detection.

Our findings in terms of clinically acceptable results were in agreement with many other software programs' results. For example, Farzan et al. [18] compared Dolphin versus Ceph Ninja programs on 30 lateral cephalograms. Uysal et al. [19] examined intraobserver and inter-observer reliability of the Dolphin software versus conventional tracing. According to them, albeit the Dolphin program could not effectively decrease intra-observer and inter-observer errors, it was much faster than the conventional method [19]. According to another study [16], in spite of some differences between the OnyxCeph program and hand-tracing technique, statistical differences were minimal and observed in only 9 out of the 18 assessed measurements. They considered OnyxCeph an efficient method to replace the conventional method [16]. Davoudian [20] compared the results of tracing using OnyxCeph program versus conventional tracing, in 30 lateral cephalographs. Tikku et al. [21] compared cephalometric analyses of 40 lateral cephalograms performed by a computer program versus the conventional method and found many statistically different results but almost no clinical difference between the two. Although the results of the whole literature have been controversial, previous studies have indicated that cephalometric programs can be reliable at least in terms of some cephalometric measurements, if not all of them. The chief reason for errors in cephalometric measurements seems to be erroneous landmark identification [16, 17, 24]. Moreover, since the Frankfurt horizontal plane has a high rate of erroneous detections [16], measurements involving this plane are more likely to become problematic [16, 24]. Some argue that cephalometric errors are not linked to the method of tracing (i.e., conventional or digital) but instead, to the types of measured variables, whether they are linear or angular [16, 21, 25]. According to them, linear measurements are more prone to errors compared to angular ones, perhaps because of image distortions [16, 22] and since image calibrations might influence angular measurements less than linear measurements [26].

This pilot study was limited by some factors. Although the sample was adequately large, it would be better if the sample size were predetermined based on calculations. Moreover, it would be better to evaluate the landmarking and tracing errors on a set of more landmarks and cephalometric tracings.

## 5. Conclusion

• In this study, all the tracing errors were smaller than the clinically acceptable limits, and hence appropriate.
• Moreover, despite more stringent thresholds set for the evaluation of cephalometric tracing accuracies, most simple errors were close to zero, and many of the very few statistically significant ones were below the stringent criterion of 1/100 of the mean of gold standard (and of course, all the 51 errors were below the clinically acceptable limit).
• The directionless absolute errors were all below 2 mm or 2°; additionally, in the case of tracings done on the computer screen versus the gold standard, absolute errors were either below the 1/100 of absolute of the gold standard means or at the level of those means.
• Finally, the intraobserver reliabilities were high. All of this suggests that manual tracing using this new software program may be accurate and reproducible.

## Figures and Tables

**Table 1:** Descriptive statistics for tracing measurements performed via the three methods. The number of samples per row is 101 (*n* = 101).

**Measurement**	**Everything on paper**		**Everything on screen**		**Landmarking on paper**	
	**Mean**	**SD**	**Mean**	**SD**	**Mean**	**SD**
N-A-Pog	−5.479	6.864	−5.488	6.879	−5.484	6.774
Facial angle	87.793	3.806	87.674	3.732	87.691	3.737
A-B plane	−6.357	3.590	−6.366	3.587	−6.293	3.704
MP angle	24.552	5.109	24.556	5.100	24.590	5.151
NSG	110.021	3.979	110.123	3.886	110.092	3.915
OP cant	7.850	4.046	7.862	4.027	7.931	4.124
Interincisal angle	126.262	10.051	126.206	10.148	126.556	9.935
L1 ^ OP	113.563	7.318	113.543	7.342	113.354	7.520
L1 ^ MP	83.370	7.636	83.393	7.673	83.551	7.794
U1 protrusion	5.275	2.396	5.276	2.390	5.978	2.725
SNA	79.634	3.330	79.692	3.233	79.653	3.246
SNB	76.263	3.675	76.241	3.591	76.210	3.637
ANB	−3.447	3.059	−3.451	3.050	−3.443	3.014
OP-S-N	18.121	4.457	18.144	4.473	18.262	4.599
MP-S-N	35.070	5.780	35.079	5.775	35.167	5.836
U1-N-A	22.394	7.167	22.416	7.185	22.175	7.102
L1-N-B	27.957	6.614	27.927	6.587	27.826	6.722

*Note:* Everything on paper refers to the fully conventional gold standard, where both landmarking and tracing have been performed conventionally on the tracing paper. Landmarking on paper refers to the semidigital method, in which landmarking is done on paper, and then, landmark coordinates are used by the program to calculate the measurements. Everything on screen refers to the fully digital method, in which the landmarking was done by the operator on the screen, and from their coordinates, the software calculated the measurements.

Abbreviations: OP, occlusal plane; SD, standard deviation.

**Table 2:** Descriptive statistics and 95% CIs for mean simple errors calculated as the gold standard (all procedures done on paper) minus semi digital (landmarking on paper but measurements done using the software). The *p* values are calculated once by comparing the mean errors with zero, and if significant, then once more by comparing them with 1/100 of the means of gold standard tracing values. All measurements were significantly smaller than 2 (all *p* values < 0.0005). The number of samples per row is 101 (*n* = 101).

**Measurement**	**Mean**	**SD**	**Min**	**Q1**	**Med**	**Q3**	**Max**	**95% CI**		**P**_0_	**P**_**g** **o** **l** **d**/100_
N-A-Pog	0.004	0.700	−1.661	−0.535	−0.059	0.633	1.474	−0.134	0.143	0.949	—
Facial angle	0.103	0.581	−1.078	−0.336	0.133	0.478	1.712	−0.012	0.217	0.079	—
A-B plane	−0.065	0.628	−1.395	−0.616	−0.057	0.428	1.025	−0.189	0.059	0.303	—
MP angle	−0.037	0.450	−1.388	−0.339	−0.051	0.321	1.059	−0.126	0.052	0.408	—
NSG	−0.072	0.709	−1.436	−0.634	−0.166	0.466	1.742	−0.212	0.068	0.312	—
OP cant	−0.080	0.654	−1.743	−0.488	−0.092	0.349	1.248	−0.209	0.049	0.221	—
Interincisal angle	−0.293	1.315	−3.639	−1.114	−0.085	0.631	2.873	−0.553	−0.034	0.027	< 0.00000005
L1 ^ OP	0.209	1.204	−3.823	−0.592	0.231	1.018	2.606	−0.028	0.447	0.084	—
L1 ^ MP	−0.180	1.207	−3.243	−0.898	−0.220	0.519	3.693	−0.418	0.058	0.137	—
U1 protrusion	−0.703	0.434	−1.679	−0.996	−0.693	−0.422	0.512	−0.789	−0.617	< 0.00000005	< 0.00000005
SNA	−0.019	0.663	−1.653	−0.478	0.067	0.470	1.623	−0.150	0.112	0.770	—
SNB	0.054	0.539	−1.223	−0.381	0.085	0.375	1.655	−0.053	0.160	0.318	—
ANB	−0.003	0.349	−0.616	−0.279	−0.005	0.265	0.830	−0.072	0.066	0.930	—
OP-S-N	−0.142	0.665	−1.797	−0.630	−0.117	0.190	1.528	−0.273	−0.010	0.035	0.0000040
MP-S-N	−0.097	0.510	−1.481	−0.485	−0.065	0.303	0.910	−0.197	0.004	0.059	—
U1-N-A	0.219	0.973	−1.959	−0.333	0.314	0.910	2.699	0.027	0.411	0.026	0.962
L1-N-B	0.131	1.097	−3.972	−0.439	0.176	0.777	2.567	−0.085	0.348	0.231	—

*Note:* The reference values for comparisons for the second *p* value column are as follows: −0.055, 0.878, −0.064, 0.246, 1.100, 0.079, 1.263, 1.136, 0.834, 0.053, 0.796, 0.763, −0.034, 0.181, 0.351, 0.224, and 0.280, respectively, for N-A-Pog, facial angle, A-B plane, MP angle, NSG, the cant of OP, Interincisal Angle, L1 ^ OP, L1 ^ MP, U1 protrusion, SNA, SNB, ANB, OP-S-N, MP-S-N, U1-N-A, and L1-N-B.

Abbreviations: CI, confidence interval based on the t distribution; Max, maximum; Med, median; Min, minimum; OP, occlusal plane; Q1, first quartile; Q3, third quartile; SD, standard deviation.

**Table 3:** Descriptive statistics and 95% CIs for mean simple errors calculated as the gold standard (all procedures done on paper) minus fully digital (both landmarking and measurements done manually on the computer). The *p* values are calculated once by comparing the mean errors with zero, and if significant, then once more by comparing them with 1/100 of the means of gold standard tracing values. All measurements were significantly smaller than 2 (all *p* values < 0.0005). The number of samples per row is 101 (*n* = 101).

**Measurement**	**Mean**	**SD**	**Min**	**Q1**	**Med**	**Q3**	**Max**	**95% CI**		**P**_0_	**P**_**g** **o** **l** **d**/100_
N-A-Pog	0.009	0.064	−0.169	−0.036	0.007	0.048	0.178	−0.004	0.021	0.165	—
Facial Angle	0.119	0.462	−0.856	−0.204	0.201	0.500	0.881	0.028	0.210	0.011	< 0.00000005
A-B Plane	0.008	0.046	−0.152	−0.019	0.012	0.038	0.133	−0.001	0.017	0.072	—
MP Angle	−0.003	0.150	−0.306	−0.113	0.006	0.116	0.303	−0.033	0.026	0.835	—
NSG	−0.103	0.642	−1.079	−0.683	−0.183	0.442	1.048	−0.229	0.024	0.111	—
OP cant	−0.011	0.060	−0.230	−0.051	−0.015	0.026	0.123	−0.023	0.001	0.064	—
Interincisal Angle	0.057	0.708	−1.346	−0.510	0.092	0.676	1.315	−0.083	0.197	0.423	—
L1 ^ OP	0.020	0.672	−1.104	−0.622	0.078	0.515	1.128	−0.112	0.153	0.763	—
L1 ^ MP	−0.022	0.516	−0.992	−0.427	−0.092	0.415	0.877	−0.124	0.080	0.666	—
U1 Protrusion	−0.001	0.046	−0.095	−0.037	−0.001	0.032	0.108	−0.010	0.008	0.861	—
SNA	−0.058	0.479	−0.772	−0.488	−0.075	0.299	0.811	−0.153	0.036	0.224	—
SNB	0.023	0.424	−0.732	−0.353	0.004	0.336	0.742	−0.061	0.106	0.591	—
ANB	0.005	0.051	−0.133	−0.030	0.001	0.037	0.195	−0.005	0.015	0.348	—
OP-S-N	−0.023	0.105	−0.250	−0.087	−0.026	0.050	0.182	−0.044	−0.002	0.032	< 0.00000005
MP-S-N	−0.009	0.217	−0.417	−0.172	−0.035	0.169	0.441	−0.052	0.034	0.681	—
U1-N-A	−0.022	0.134	−0.396	−0.105	−0.012	0.070	0.290	−0.048	0.005	0.108	—
L1-N-B	0.030	0.170	−0.374	−0.097	0.032	0.160	0.339	−0.003	0.064	0.078	—

*Note:* The reference values for comparisons for the second *p* value column are as follows: −0.055, 0.878, −0.064, 0.246, 1.100, 0.079, 1.263, 1.136, 0.834, 0.053, 0.796, 0.763, −0.034, 0.181, 0.351, 0.224, and 0.280, respectively, for N-A-Pog, facial angle, A-B plane, MP angle, NSG, the cant of OP, interincisal angle, L1 ^ OP, L1 ^ MP, U1 protrusion, SNA, SNB, ANB, OP-S-N, MP-S-N, U1-N-A, and L1-N-B.

Abbreviations: CI, confidence interval based on the t distribution; Max, maximum; Med, median; Min, minimum; OP, occlusal plane; Q1, first quartile; Q3, third quartile; SD, standard deviation.

**Table 4:** Descriptive statistics and 95% CIs for mean simple errors calculated as semidigital (landmarking on paper but calculations by computer) minus fully digital (both landmarking and measurements done manually on the computer). The *p* values are calculated once by comparing the mean errors with zero and, if significant, then once more by comparing them with 1/100 of the means of gold standard tracing values. All measurements were significantly smaller than 2 (all *p* values < 0.0005). The number of samples per row is 101 (*n* = 101).

**Measurement**	**Mean**	**SD**	**Min**	**Q1**	**Med**	**Q3**	**Max**	**95% CI**		**P**_0_	**P**_**g** **o** **l** **d**/100_
N-A-Pog	0.004	0.696	−1.433	−0.627	0.091	0.555	1.600	−0.133	0.142	0.949	—
Facial angle	0.016	0.360	−0.967	−0.185	0.025	0.281	0.960	−0.055	0.087	0.652	—
A-B plane	0.073	0.627	−1.009	−0.412	0.059	0.615	1.422	−0.051	0.197	0.245	—
MP angle	0.034	0.414	−1.001	−0.273	0.029	0.334	1.160	−0.048	0.116	0.410	—
NSG	−0.031	0.362	−0.828	−0.287	−0.039	0.220	0.871	−0.102	0.040	0.392	—
OP cant	0.069	0.657	−1.352	−0.328	0.095	0.508	1.729	−0.061	0.199	0.294	—
Interincisal angle	0.350	1.231	−3.439	−0.278	0.388	1.207	3.023	0.107	0.593	0.005	< 0.00000005
L1 ^ OP	−0.189	1.148	−2.723	−0.957	−0.370	0.424	4.188	−0.416	0.038	0.101	—
L1 ^ MP	0.158	1.070	−3.829	−0.412	0.209	0.874	2.286	−0.053	0.369	0.141	—
U1 protrusion	0.702	0.436	−0.443	0.427	0.704	1.012	1.683	0.616	0.788	< 0.00000005	< 0.00000005
SNA	−0.039	0.514	−1.290	−0.466	−0.018	0.248	1.153	−0.141	0.063	0.448	—
SNB	−0.031	0.403	−1.039	−0.326	−0.073	0.215	0.804	−0.111	0.048	0.440	—
ANB	0.008	0.351	−0.767	−0.273	0.046	0.275	0.636	−0.061	0.077	0.822	—
OP-S-N	0.119	0.641	−1.508	−0.181	0.114	0.579	1.764	−0.008	0.245	0.065	—
MP-S-N	0.088	0.458	−0.995	−0.265	0.110	0.365	1.358	−0.003	0.178	0.057	—
U1-N-A	−0.241	0.964	−2.538	−0.953	−0.310	0.317	1.962	−0.431	−0.051	0.014	0.0000046
L1-N-B	−0.101	1.075	−2.535	−0.769	−0.152	0.492	3.705	−0.314	0.111	0.346	—

*Note:* The reference values for comparisons for the second *p* value column are as follows: −0.055, 0.878, −0.064, 0.246, 1.100, 0.079, 1.263, 1.136, 0.834, 0.053, 0.796, 0.763, −0.034, 0.181, 0.351, 0.224, and 0.280, respectively, for N-A-Pog, facial angle, A-B plane, MP angle, NSG, the cant of OP, interincisal angle, L1 ^ OP, L1 ^ MP, U1 protrusion, SNA, SNB, ANB, OP-S-N, MP-S-N, U1-N-A, and L1-N-B.

Abbreviations: CI, confidence interval based on the t distribution; Max, maximum; Med, median; Min, minimum; OP, occlusal plane; Q1, first quartile; Q3, third quartile; SD, standard deviation.

**Table 5:** Descriptive statistics and 95% CIs for mean directionless errors calculated as the absolute value of the gold standard (all on paper) minus semidigital (landmarking on paper but measurements using the computer). The *p* values are calculated once by comparing the mean errors with zero, and if significant, then once more by comparing them with the absolute values of 1/100 of the means of gold standard tracing values. All measurements were significantly smaller than 2 (all *p* values < 0.0005). The number of samples per row is 101 (*n* = 101).

**Measurement**	**Mean**	**SD**	**Min**	**Q1**	**Med**	**Q3**	**Max**	**95% CI**		**P**_0_	**P**_**g** **o** **l** **d**/100_
N-A-Pog	0.587	0.378	0.000	0.269	0.540	0.835	1.661	0.512	0.661	< 0.00000005	< 0.00000005
Facial angle	0.477	0.344	0.009	0.207	0.432	0.711	1.712	0.409	0.545	< 0.00000005	< 0.00000005
A-B plane	0.531	0.337	0.024	0.222	0.479	0.770	1.395	0.465	0.598	< 0.00000005	< 0.00000005
MP angle	0.367	0.260	0.008	0.171	0.333	0.518	1.388	0.316	0.419	< 0.00000005	0.0000079
NSG	0.604	0.373	0.007	0.308	0.557	0.867	1.742	0.530	0.677	< 0.00000005	< 0.00000005
OP cant	0.524	0.396	0.012	0.194	0.407	0.789	1.743	0.446	0.602	< 0.00000005	< 0.00000005
Interincisal angle	1.056	0.832	0.003	0.390	0.912	1.494	3.639	0.892	1.220	<0.00000005	0.014
L1 ^ OP	0.968	0.739	0.010	0.374	0.807	1.418	3.823	0.823	1.114	< 0.00000005	0.025
L1 ^ MP	0.954	0.755	0.022	0.344	0.787	1.486	3.693	0.805	1.103	< 0.00000005	0.111
U1 protrusion	0.728	0.390	0.025	0.424	0.693	0.996	1.679	0.651	0.805	< 0.00000005	< 0.00000005
SNA	0.537	0.385	0.001	0.237	0.478	0.801	1.653	0.461	0.613	< 0.00000005	< 0.00000005
SNB	0.432	0.324	0.005	0.160	0.376	0.681	1.655	0.368	0.496	< 0.00000005	< 0.00000005
ANB	0.292	0.189	0.005	0.124	0.268	0.432	0.830	0.255	0.330	< 0.00000005	< 0.00000005
OP-S-N	0.537	0.414	0.000	0.189	0.430	0.803	1.797	0.455	0.618	< 0.00000005	<0.00000005
MP-S-N	0.422	0.299	0.001	0.181	0.405	0.588	1.481	0.363	0.481	< 0.00000005	0.019
U1-N-A	0.805	0.584	0.044	0.324	0.683	1.135	2.699	0.690	0.921	< 0.00000005	< 0.00000005
L1-N-B	0.844	0.708	0.001	0.311	0.682	1.206	3.972	0.705	0.984	< 0.00000005	< 0.00000005

*Note:* The reference values for comparisons for the second *p* value column are as follows: 0.055, 0.878, 0.064, 0.246, 1.100, 0.079, 1.263, 1.136, 0.834, 0.053, 0.796, 0.763, 0.034, 0.181, 0.351, 0.224, and 0.280, respectively, for N-A-Pog, facial angle, A-B plane, MP angle, NSG, the cant of OP, interincisal angle, L1 ^ OP, L1 ^ MP, U1 protrusion, SNA, SNB, ANB, OP-S-N, MP-S-N, U1-N-A, and L1-N-B.

Abbreviations: CI, confidence interval based on the t distribution; Max, maximum; Med, median; Min, minimum; OP, occlusal plane; Q1, first quartile; Q3, third quartile; SD, standard deviation.

**Table 6:** Descriptive statistics and 95% CIs for mean directionless errors calculated as the absolute value of the gold standard (all on paper) minus fully digital (both landmarking and measurements done manually on the computer). The *p* values are calculated once by comparing the mean errors with zero and, if significant, then once more by comparing them with the absolute values of 1/100 of the means of gold standard tracing values. All measurements were significantly smaller than 2 (all *p* values < 0.0005). The number of samples per row is 101 (*n* = 101).

**Measurement**	**Mean**	**SD**	**Min**	**Q1**	**Med**	**Q3**	**Max**	**95% CI**		**P**_0_	**P**_**g** **o** **l** **d**/100_
N-A-Pog	0.050	0.040	0.000	0.021	0.041	0.070	0.178	0.042	0.058	< 0.00000005	0.251
Facial angle	0.406	0.248	0.000	0.204	0.391	0.591	0.881	0.357	0.455	< 0.00000005	< 0.00000005
A-B plane	0.036	0.029	0.000	0.014	0.030	0.050	0.152	0.031	0.042	< 0.00000005	< 0.00000005
MP angle	0.126	0.080	0.000	0.058	0.115	0.190	0.306	0.110	0.142	< 0.00000005	< 0.00000005
NSG	0.568	0.311	0.010	0.325	0.551	0.835	1.079	0.506	0.629	< 0.00000005	< 0.00000005
OP cant	0.048	0.038	0.001	0.019	0.041	0.067	0.230	0.040	0.055	< 0.00000005	< 0.00000005
Interincisal angle	0.613	0.355	0.038	0.323	0.582	0.913	1.346	0.542	0.683	< 0.00000005	< 0.00000005
L1 ^ OP	0.582	0.332	0.017	0.283	0.565	0.900	1.128	0.516	0.648	< 0.00000005	< 0.00000005
L1 ^ MP	0.439	0.269	0.004	0.191	0.427	0.674	0.992	0.386	0.492	< 0.00000005	< 0.00000005
U1 protrusion	0.038	0.026	0.000	0.018	0.036	0.056	0.108	0.033	0.043	< 0.00000005	0.0000002
SNA	0.419	0.237	0.000	0.205	0.437	0.641	0.811	0.372	0.465	< 0.00000005	< 0.00000005
SNB	0.362	0.218	0.004	0.170	0.353	0.551	0.742	0.319	0.405	< 0.00000005	< 0.00000005
ANB	0.040	0.033	0.001	0.018	0.033	0.052	0.195	0.033	0.046	< 0.00000005	0.106
OP-S-N	0.088	0.062	0.001	0.036	0.072	0.136	0.250	0.075	0.100	< 0.00000005	< 0.00000005
MP-S-N	0.184	0.114	0.002	0.086	0.172	0.266	0.441	0.161	0.206	< 0.00000005	< 0.00000005
U1-N-A	0.107	0.082	0.000	0.046	0.093	0.155	0.396	0.091	0.124	< 0.00000005	< 0.00000005
L1-N-B	0.142	0.097	0.000	0.056	0.136	0.206	0.374	0.123	0.161	< 0.00000005	< 0.00000005

*Note:* The reference values for comparisons for the second *p* value column are as follows: 0.055, 0.878, 0.064, 0.246, 1.100, 0.079, 1.263, 1.136, 0.834, 0.053, 0.796, 0.763, 0.034, 0.181, 0.351, 0.224, and 0.280, respectively, for N-A-Pog, facial angle, A-B plane, MP angle, NSG, the cant of OP, interincisal angle, L1 ^ OP, L1 ^ MP, U1 protrusion, SNA, SNB, ANB, OP-S-N, MP-S-N, U1-N-A, and L1-N-B.

Abbreviations: CI, confidence interval based on the t distribution; Max, maximum; Med, median; Min, minimum; OP, occlusal plane; Q1, first quartile; Q3, third quartile; SD, standard deviation.

**Table 7:** Descriptive statistics and 95% CIs for mean directionless errors calculated as the absolute value of semidigital (landmarking on paper but calculations by computer) minus fully digital (both landmarking and measurements done manually on the computer screen). The *p* values are calculated once by comparing the mean errors with zero and, if significant, then once more by comparing them with the absolute values of 1/100 of the means of gold standard tracing values. All measurements were significantly smaller than 2 (all *p* values < 0.0005). The number of samples per row is 101 (*n* = 101).

**Measurement**	**Mean**	**SD**	**Min**	**Q1**	**Med**	**Q3**	**Max**	**95% CI**		**P**_0_	**P**_**g** **o** **l** **d**/100_
N-A-Pog	0.586	0.371	0.001	0.282	0.557	0.835	1.600	0.513	0.659	< 0.00000005	< 0.00000005
Facial angle	0.282	0.223	0.002	0.107	0.236	0.382	0.967	0.238	0.326	< 0.00000005	< 0.00000005
A-B plane	0.532	0.337	0.012	0.247	0.487	0.784	1.422	0.466	0.598	< 0.00000005	< 0.00000005
MP angle	0.338	0.239	0.002	0.140	0.321	0.472	1.160	0.291	0.386	< 0.00000005	0.00017
NSG	0.296	0.209	0.013	0.127	0.252	0.426	0.871	0.254	0.337	< 0.00000005	< 0.00000005
OP cant	0.526	0.397	0.021	0.206	0.401	0.770	1.729	0.447	0.604	< 0.00000005	< 0.00000005
Interincisal angle	1.014	0.774	0.023	0.332	0.810	1.512	3.439	0.861	1.167	< 0.00000005	0.0016988
L1 ^ OP	0.932	0.691	0.025	0.424	0.808	1.283	4.188	0.795	1.068	< 0.00000005	0.004
L1 ^ MP	0.855	0.658	0.046	0.320	0.704	1.293	3.829	0.725	0.985	< 0.00000005	0.748
U1 protrusion	0.724	0.399	0.006	0.438	0.704	1.012	1.683	0.645	0.803	< 0.00000005	< 0.00000005
SNA	0.417	0.301	0.005	0.164	0.390	0.612	1.290	0.357	0.476	< 0.00000005	< 0.00000005
SNB	0.325	0.237	0.001	0.130	0.276	0.531	1.039	0.279	0.372	< 0.00000005	< 0.00000005
ANB	0.297	0.185	0.018	0.139	0.275	0.435	0.767	0.261	0.334	< 0.00000005	< 0.00000005
OP-S-N	0.505	0.409	0.003	0.160	0.361	0.754	1.764	0.424	0.585	< 0.00000005	< 0.00000005
MP-S-N	0.382	0.264	0.021	0.173	0.337	0.519	1.358	0.330	0.434	< 0.00000005	0.230
U1-N-A	0.803	0.579	0.008	0.317	0.723	1.194	2.538	0.689	0.918	< 0.00000005	< 0.00000005
L1-N-B	0.833	0.683	0.010	0.327	0.674	1.186	3.705	0.698	0.968	< 0.00000005	< 0.00000005

*Note:* The reference values for comparisons for the second *p* value column are as follows: 0.055, 0.878, 0.064, 0.246, 1.100, 0.079, 1.263, 1.136, 0.834, 0.053, 0.796, 0.763, 0.034, 0.181, 0.351, 0.224, and 0.280, respectively, for N-A-Pog, facial angle, A-B plane, MP angle, NSG, the cant of OP, interincisal angle, L1 ^ OP, L1 ^ MP, U1 protrusion, SNA, SNB, ANB, OP-S-N, MP-S-N, U1-N-A, and L1-N-B.

Abbreviations: CI, confidence interval based on the t distribution; Max, maximum; Med, median; Min, minimum; OP, occlusal plane; Q1, first quartile; Q3, third quartile; SD, standard deviation.

## Data Availability

The data are available from the authors upon request.
